# Towards objective measurement of reproductive performance of traditionally managed goat flocks in the drylands of Ethiopia

**DOI:** 10.1007/s11250-021-02556-y

**Published:** 2021-02-08

**Authors:** Gezahegn Alemayehu, Gezahegne Mamo, Biruk Alemu, Hiwot Desta, Barbara Wieland

**Affiliations:** 1 International Livestock Research Institute (ILRI), P.O. Box: 5689, Addis Ababa, Ethiopia; 2 Department of Microbiology, Immunology and Veterinary Public Health, College of Veterinary Medicine and Agriculture, Addis Ababa University, Bishoftu, Ethiopia; 3 College of Veterinary Medicine, Samara University, Samara, Ethiopia

**Keywords:** Goat reproductive index, Kidding interval, Reproductive output, Reproductive wastage

## Abstract

Reproductive performance is a key determinant for the efficiency of goat production. Regular monitoring of reproductive efficiency is essential to assess management and to avoid financial losses due to poor performance. To allow more objective measurement and comparisons over time, we propose a novel quantitative approach for defining annual reproductive performance by combining common performance indicators into a goat flock index. Commonly used reproductive performance measures were collected from 242 goat flocks in four districts in dryland of Ethiopia between July 2018 and February 2019. Principal component analysis (PCA) was performed to identify biologically meaningful latent components that explain annual reproductive output (ARO) and annual reproductive wastage (ARW). Together with the remaining annual reproductive performance measures, the ARO and ARW components were included in a PCA to derive an algorithm for a goat annual reproductive performance index (G-ARPI). One component representing variation in kidding interval, PC_ARO_1 and PC_ARW_1 was extracted and normalized to a 10-scale value. The flocks were classified into good performing (15.63%) with index > 8.5, moderately performing (48.21%) with index values ranging from 6.5 to 8.5 and poor performing (36.16%) with index < 6.5. Good performing flocks have higher scores for reproductive output measures, lower scores for reproductive wastage and lower kidding interval. The proposed G-ARPI can be used as an objective tool to compare reproductive performance between management systems, evaluate the costs of poor reproductive management and will be useful for economic models that aim to identify the most cost-efficient intervention option and monitor the impact of interventions. We present here the index for goat production in dryland systems in Ethiopia; the approach can easily be adapted to other production systems elsewhere.

**Supplementary Information:**

The online version contains supplementary material available at 10.1007/s11250-021-02556-y.

## Introduction

Goat production under extensive low-input systems plays an important role in ensuring food security and supporting rural livelihoods in arid and semi-arid areas where conditions for crop farming are limited (Muigai et al. 2017; Pulina et al. 2017). In these systems, goats are an integral part of households, providing nutrition, employment and easily accessible sources of income (Ørskov 2011; Hassen and Tesfaye 2014). Goats can survive in harsh environmental conditions and benefit from feed resource which is not used by other ruminants (Nardone et al. 2010; Gaughan et al. 2018). At national level, they are an important source of foreign currency through live animal and meat export (FAOSTAT 2019).

However, low productivity per animal and flock limits the potential contribution of goats for rural households in the arid areas of Ethiopia (Solomon et al. 2014; Feldt et al. 2016). Low productivity of the flock is linked to poor nutritional status due to poor pasture quantity and quality or lack of feed and high burden of disease, which together contribute to reproductive failure and poor growth rates (Mayberry et al. 2018).

Reproductive performance is a key determinant of the efficiency of goat production (Delgadillo and Martin 2015). Accordingly, regular monitoring of reproductive efficiency is essential to assess management and often acts as an early indicator for health problems and thus, helps to avoid financial losses due to poor performance (FAO 1993). Several parameters are commonly used to measure reproductive performance, with the most common being fertility (the proportion of pregnant does exposed or mated to the buck), kidding percentage (number of kids born per doe exposed to the buck), prolificacy (the proportion of kids born alive), abortion rate (proportion of premature born kids), age at first kidding, kidding interval (interval between successive kidding), and weaning percentage (percentage of kids weaned per doe exposed to the buck). Kid losses are usually calculated as stillbirth rates and preweaning mortality rates as a proportion of kids weaned and kids born alive (Galina et al. 1995; Mellado et al. 2006; Song et al. 2006).

In Ethiopia, researches undertaken on goat reproductive performance were largely based on single trait, often used as part of a breed performance evaluation program on research stations (Belay et al. 2014; Solomon et al. 2014; Deribe et al. 2015). However, technological solutions developed through conventional station-based agricultural research have failed to achieve the expected results in the small-scale farming sector of the developing world (Stroud et al. 2000). The goals for reproductive performance vary tremendously between different goat production systems and need to take into account the management systems and constraints at the flock level. The reproductive performance should be measured by obtaining an overall picture of the flock’s reproductive performance preferably considers various individual components of reproductive activities and integrating them into an index. The minimum measures that should be included in an integrated index for annual flock performance are average age at first kidding, kidding interval, annual reproductive output and annual reproductive wastage (Wilson 1989; Ibrahim 1998; Browning et al. 2011).

Increasing flock reproductive performance can be achieved through different interventions, including better managemental practice, nutrition, genetics and healthcare adopted by the producers and extension agents (Mayberry et al. 2018). To ensure cost-efficiency of interventions, the resulting reproductive performance change should be measured appropriately and objectively in a way that can provide a comprehensive picture of past reproductive performance, current changes and future expectations.

This paper assesses commonly used measures for goat flock reproductive performance in goats in dryland systems in Ethiopia and proposes a novel quantitative approach for defining annual reproductive performance at flock level by combining performance indicators into a goat specific index.

## Material and methods

### Study sites

**Fig. 1:**
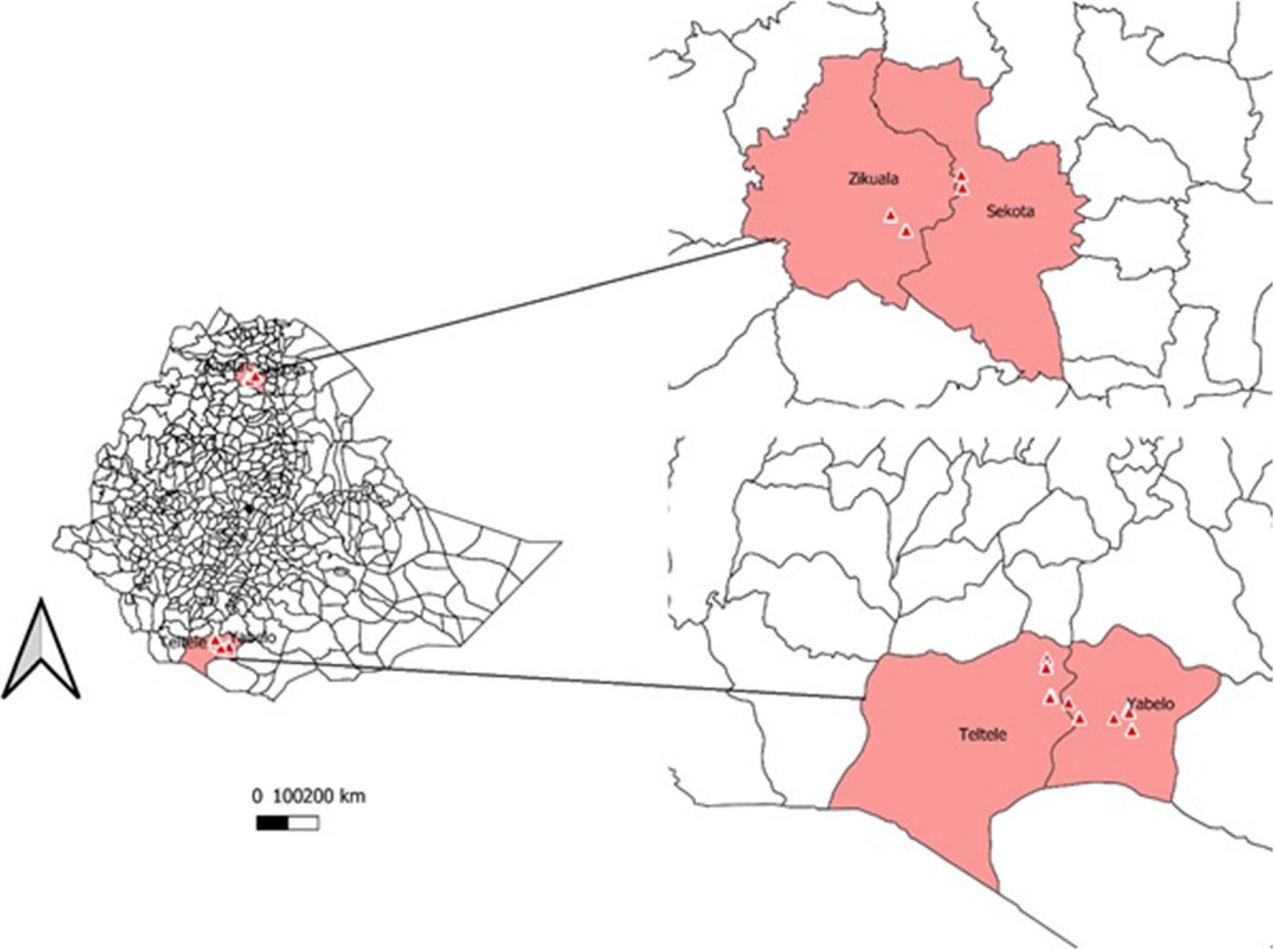
Map of Ethiopia showing the study districts

This study was conducted in four locations (districts) in two regional states—Ziquala and Abergele in Amhara Region and Yabello and Elwaya in Oromia region (Fig. 1). Ziquala and Abergele represent the lowland mixed crop-livestock production system in northern part of Ethiopia and Yabello and Elwaya represent the pastoral production system in southern part of Ethiopia (Table 1). The sites are part of the CGIAR research program on livestock (CRP Livestock) and had been selected based on agro-ecologies and production systems, potential of the areas for goat production, accessibility and willingness of the community to participate in further studies and importance of sheep and goats to household livelihoods (Haile et al. 2019). Two kebeles (=smallest administrative unit in Ethiopia) were selected in each of the four districts. One of these kebeles was an active CRP livestock research site and one kebele had not seen any previous interventions. The CRP livestock intervention *kebeles* had received animal health interventions such as vaccination for Pasteurellosis, Contagious caprine pleuropneumonia, Peste des petits ruminants and goat pox and were involved in community-based internal parasite control programs. They also had received animal health training on the following topics: (1) integrated herd health approach to reduce the impact of respiratory disease in small ruminants, (2) causes of reproductive health problems in small ruminants and possible control options and (3) community-based strategic internal parasite control in small ruminants. They were also a member of a community-based breeding program.

**Table 1:** Description of study areas in Ethiopia

No.	District	Production system	Village	Intervention status	Altitude (masl)	Rainfall (mm)	Average temperature (°C)
1	Abergele	Lowland, mixed crop livestock	Sazba	CRP intervention	1348	647	24
			Belteharf	Control			
2	Ziquala	Lowland, mixed crop-livestock	Bilaqu	CRP intervention	1486	732	22
			Tsitsika	Control			
3	Yabello	Lowlands, pastoral	Derito	CRP intervention	1588	625	20
			Dida Yabello	Control			
4	Elwaya	Lowlands, pastoral	Adegalchet	CRP intervention	1181	493	22
			Chari	Control			

*CRP* CGIAR research program

### Study households and flocks

In each kebele, 32 households were selected randomly, in total 242 households. Lists of households for each kebele (smallholders’ farmers/pastoralists in the study sites who own small ruminants) were obtained from enumerators of the CRP livestock intervention *kebeles* and from key informants at control *kebele* 1 day before the survey. From these lists, households were selected randomly using the random function in Excel. Facilitators then contacted the household heads, asked for their willingness to participate and planned the timing of the interview. Only households who own goats were considered in this study.

### Data collection processes

Data were collected through a face-to-face structured interview using a questionnaire. Questions were designed based on a literature review and the experiences of researchers. To prevent ambiguity and obtain concise information, mainly closed questions were used. The questionnaire was pre-tested on a pilot group of 15 farmers who were not included in the study population and necessary adjustments were made after the pre-testing. The questions were coded using Epi Info™ 7.2.1.0 software and copied onto Galaxy Tab A (2016) for digital data collection. The recorded responses were transferred and stored on a personal laptop computer and subsequently exported to statistical software GeoDa (https://geodacenter.github.io/) where primary processing and quality checks were undertaken.

The interviews were conducted by four trained veterinarian and/or animal production experts from the National Agricultural research system who spoke the local language of the respective study sites. They received training on the interview tool, interview approach and digital recording of responses. The training ensured a common understanding of the meaning of each question and in what way to ask participants.

Interviews were conducted between July 2018 and February 2019 in a place where both interviewer and participant felt comfortable. The farmers/pastoralists were informed about the purpose of the study and approximate time the interview will take, and their oral informed consent was sought before their participation in the survey.

### Reproductive performance measure calculation

Reproductive performance measures included in the data collection were age at first kidding, kidding interval, number of pregnancy, number of kids born, number of kids survived to wean, number of abortion, number of defective birth (dystocia, retained placenta, weak kids, defective kids), number of kids died before weaning (3 months), and number of kid per doe lifetime. Each reproductive performance measure was estimated by farmers/pastoralists based on their information on reproductive events in the flocks during 1 year before the interview. The number of offspring produced per doe lifetime in each flock was estimated based on information on offspring produced from at least two culled/dead does in the last 5-year period. Respondents were also asked about their confidence in their estimation. If they were not confident about their estimates, the data entry was left empty and treated as missing. Each reproductive performance measure was estimated at flock level based on farmers/pastoralists information and a separate data table was created in the database. The following reproduction traits were calculated in each flock as

$$ {\displaystyle \begin{array}{c}\mathrm{Age}\ \mathrm{at}\ \mathrm{first}\ \mathrm{kidding}=\frac{\sum \mathrm{age}\ \mathrm{at}\ \mathrm{first}\ \mathrm{kidding}\ \mathrm{in}\ \mathrm{months}\kern0.75em }{\sum \mathrm{does}\ \mathrm{kidded}\ \mathrm{for}\ \mathrm{first}\ \mathrm{time}\kern0.5em \mathrm{in}\ \mathrm{the}\ \mathrm{flock}\mathrm{s}\ 1\ \mathrm{year}\ \mathrm{before}\ \mathrm{the}\ \mathrm{survey}\kern0.5em }\\ {}\mathrm{Kidding}\ \mathrm{in}\mathrm{terval}=\frac{\sum \mathrm{months}\ \mathrm{between}\ \mathrm{successive}\ \mathrm{kidding}\ \mathrm{during}\ 1\ \mathrm{year}\ \mathrm{prior}\ \mathrm{to}\ \mathrm{the}\ \mathrm{survey}\kern0.5em }{\sum \mathrm{does}\ \mathrm{kidded}\ \mathrm{during}\ 1\ \mathrm{year}\ \mathrm{prior}\ \mathrm{to}\ \mathrm{the}\ \mathrm{survey}\ }\\ {}\begin{array}{c}\mathrm{Pregnancy}\ \mathrm{rate}=\frac{\sum \mathrm{pregnant}\ \mathrm{does}\ \mathrm{during}\ 1\ \mathrm{year}\ \mathrm{prior}\ \mathrm{to}\ \mathrm{survey}\ }{\sum \mathrm{mature}\ \mathrm{does}\ \mathrm{in}\ \mathrm{the}\ \mathrm{flock}\ \mathrm{exposed}\ \mathrm{to}\ \mathrm{buck}\ \mathrm{during}\ 1\ \mathrm{year}\ \mathrm{prior}\ \mathrm{to}\ \mathrm{survey}} \times 100\\ {}\mathrm{Annual}\ \mathrm{kidding}\ \mathrm{rate}=\frac{\sum \mathrm{kids}\ \mathrm{born}\ \mathrm{during}\ 1\ \mathrm{year}\ \mathrm{prior}\ \mathrm{to}\ \mathrm{survey}\kern0.5em }{\sum \mathrm{mature}\ \mathrm{does}\ \mathrm{in}\ \mathrm{the}\ \mathrm{flock}\ \mathrm{exposed}\ \mathrm{to}\ \mathrm{buck}\ \mathrm{during}\ 1\ \mathrm{year}\ \mathrm{prior}\ \mathrm{to}\ \mathrm{survey}} \times 100\\ {}\begin{array}{c}\mathrm{Annual}\ \mathrm{weaning}\ \mathrm{rate}=\frac{\sum \mathrm{kids}\ \mathrm{weaned}\ \mathrm{during}\ 1\ \mathrm{year}\ \mathrm{prior}\ \mathrm{to}\ \mathrm{survey}\ }{\sum \mathrm{mature}\ \mathrm{does}\ \mathrm{in}\ \mathrm{the}\ \mathrm{flock}\ \mathrm{exposed}\ \mathrm{to}\ \mathrm{buck}\ \mathrm{during}\ 1\ \mathrm{year}\ \mathrm{prior}\ \mathrm{to}\ \mathrm{survey}}\times 100\\ {}\mathrm{Abortion}\ \mathrm{rate}=\frac{\sum \mathrm{kids}\ \mathrm{lost}\ \mathrm{before}\ \mathrm{expected}\ \mathrm{parturition}\ \mathrm{during}\ 1\ \mathrm{year}\ \mathrm{prior}\ \mathrm{to}\ \mathrm{the}\ \mathrm{survey}}{\sum \mathrm{pregnant}\ \mathrm{does}\ \mathrm{in}\ \mathrm{the}\ \mathrm{flock}\ \mathrm{during}\ 1\ \mathrm{year}\ \mathrm{prior}\ \mathrm{to}\ \mathrm{the}\ \mathrm{survey}}\times 100\\ {}\begin{array}{c}\mathrm{Mortality}\ \mathrm{rate}=\frac{\sum \mathrm{kids}\ \mathrm{died}\ \mathrm{before}\ 3\ \mathrm{months}\ \mathrm{of}\ \mathrm{age}\ \mathrm{during}\ 1\ \mathrm{year}\ \mathrm{prior}\ \mathrm{to}\ \mathrm{the}\ \mathrm{survey}}{\sum \mathrm{all}\ \mathrm{kids}\ \mathrm{born}\ \mathrm{alive}\ \mathrm{in}\ \mathrm{the}\ \mathrm{flock}\ \mathrm{during}\ 1\ \mathrm{year}\ \mathrm{prior}\ \mathrm{to}\ \mathrm{the}\ \mathrm{survey}}\times 100\\ {}\mathrm{Birth}\ \mathrm{defects}\ \mathrm{rate}=\frac{\sum \mathrm{defective}\ \mathrm{births}\ \mathrm{during}\ 1\ \mathrm{year}\ \mathrm{prior}\ \mathrm{to}\ \mathrm{the}\ \mathrm{survey}}{\sum \mathrm{pregnant}\ \mathrm{does}\ \mathrm{in}\ \mathrm{the}\ \mathrm{flock}\ \mathrm{during}\ 1\ \mathrm{year}\ \mathrm{prior}\ \mathrm{to}\ \mathrm{the}\ \mathrm{survey}}\times 100\end{array}\end{array}\end{array}\end{array}} $$

### Data analysis

Flock level reproductive performance was estimated by combining data on reproductive performance traits collected from 242 flocks in the drylands of Ethiopia. First, descriptive statistics, such as mean and SD, were used to summarize data. As a first step, principal component analysis (PCA) was performed separately on data linked to annual reproductive outputs (pregnancy rate, kidding rate and weaning rate) and annual reproductive wastage (abortion rate, kid mortality rate and birth defective rate). Components with eigenvalues greater than 1 were identified and retained. As second step, the retained components from step 1 were then combined with remaining indicators (kidding interval and age at first kidding) with PCA to derive an annual reproductive performance index of flocks. In all PCAs, resulting components with eigenvalues greater than 1 were considered and only component loadings greater than 0.4 or below − 0.4 were retained in the final model. This component referred to as ‘goat annual reproductive performance index’ (G-ARPI). Statistical analyses were performed in R (R Development Core Team 2010; R Foundation for Statistical Computing, Vienna, Austria) and GeoDa. Component scores were tested for normality and transformed into a normally distributed scale using appropriate transformation to have an easily understandable and communicable measure. The resulting values of the annual reproductive performance index were then used to define three ordered groups (poor, moderate and good annual reproductive performance). In all analysis, confidence level (CI) was at 95% and *P* ≤ 0.05 was considered significant.

## Results

### Flock characteristics

Of the 242 goat flocks enrolled in the study, 114 (47.11%) were managed under a lowland mixed crop-livestock production system and 128 (52.89%) under a pastoral production system. Long-eared Somali and Abergele were the predominant goat breeds kept by pastoralists in Borena and the lowlands of Waghimira, respectively. Households kept goats for milk, meat and immediate cash income. The median size of the flocks was 12.5 breeding does with many households (40.08%) having between 10 and 20 does. Seventy-seven farms (31.82%) had less than 10 does and 68 farms (28.1%) had more than 30 does with the largest having 100 does. Only 3 flocks (1.24%) were goats only herds, while 12 farms (4.9%) managed their goat flock with at least one other livestock species and most of the farms (93.8%) managed their goat with two or more livestock species. Flocks were kept under traditional extensive management systems and therefore fully dependent on grazing lands, with overall limited inputs. The flocks were grazed freely on pastures during daytime and kept in open enclosure (74.38%) or house during the night (25.62%). All day-to-day herding decisions were made by the owner and breeding was uncontrolled. Fertile bucks were allowed to remain continuously with a group of females throughout the year.

### Reproductive performance measures

The means and standard deviations for the nine reproductive performance measures analysed in this study, aggregated by the production system, are summarized in Table 2. The overall mean age at first kidding was 16.09 (± 3.83) months. Pastoral flocks kidded at an earlier mean age (15.5 ± 3.4 months) than mixed crop-livestock flocks (16.8 ± 4.2), *P* value = 0.012. Overall mean months between successive kidding was 8.3 ± 1.9 months, with pastoral flocks having lower kidding intervals than mixed crop-livestock flocks (*P* = 0.005).

**Table 2:** Summary of goat reproductive performance parameters in dryland areas of Ethiopia

Reproductive parameters	Flock	Overall mean (±SD)	Production system		*P* value
			Crop livestock	Pastoral	
Age at first kidding, months	232	16.09(± 3.83)	16.79(± 4.18)	15.52(± 3.44)	0.012
Pregnancy rate, %	241	83.9(± 35.55)	83.16(± 43.80)	84.57 (± 26.19)	0.759
Kidding interval, month	231	8.31(± 1.968)	8.71(± 2.33)	7.98(1.54)	0.005
Kidding rate, %	241	71.54(± 42.77)	71.39(± 42.78)	74.1 (± 29.17)	0.584
Weaning rate, %	241	62.13(± 36.38)	56.0 (± 40.03)	67.57 (± 31.95)	0.000
Abortion rate, %	236	21.73(± 26.92)	19.36(24.81)	23.89(28.635)	0.197
Birth defects rate, %	240	11.06(± 20.84)	12.89(± 22.43)	9.39(19.23)	0.195
Kid mortality rate, %	239	18.17(25.54)	25.16(29.31)	11.8(19.59)	0.000
Kids per doe lifetime	231	10.22(± 3.47)	9.84(± 4.30)	10.52(± 2.60)	0.140

The average annual pregnancy rate reported in the present study for the dryland flocks was 83.9% (± 35.55). The average annual kidding and weaning rate were 71.54% (± 42.7) and 62.13% (± 36.38), respectively. Mean offspring produced per lifetime of the doe was estimated at 10.22 kids (± 3.47). Regarding reproductive failures, mean abortion, birth defects and kid mortality rates per flock were 21.73% (± 26.92), 11.06% (± 20.84) and 18.17% (25.54), respectively.

### Goat annual reproductive performance index

The annual reproductive performance index (G-ARPI) was predicted by combining reproductive performance traits.

First, two principal component analyses were performed to identify biologically meaningful latent components that explain annual reproductive output (ARO) and annual reproductive wastage (ARW) based on farmers’ information in relation to reproductive performance measures. One component with eigenvalues greater than 1, representing 77.78% and 44.88% of the common variance PC_ARO_ and PC_ARW_, was identified and retained (Table 3).

**Table 3:** Loadings of annual reproductive output and wastage on the first two principal components with eigenvalue

RP measures	PC1	PC2
Annual reproductive output (ARO) measures		
Pregnancy rate	0.53	0.80
Kidding rate	0.62	− 0.14
Weaning rate	0.58	− 0.58
Eigenvalue	2.33	0.53
% of explained variability	77.78	17. 74
Annual reproductive wastage (ARW) measures		
Abortion rate	0.64	0.17
Birth defects rate	0.49	0.83
Kid mortality rate	0.59	− 0.52
Eigenvalue	1.346	0.89
% of explained variability	44. 88	29.97

*RP* reproductive performance

**Fig. 2:**
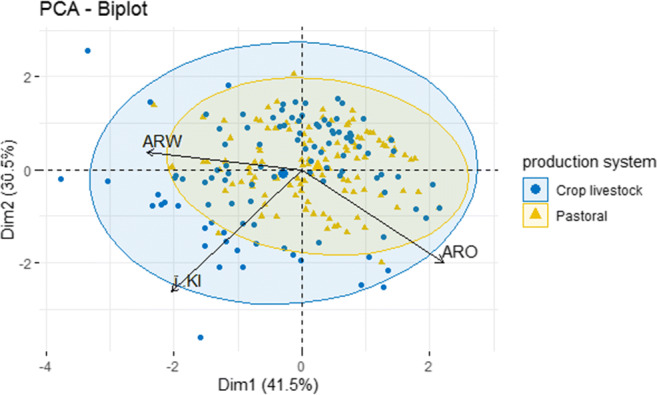
Goat flocks (dots) along the first two PCs with 95% concentration ellipses

**Fig. 3:**
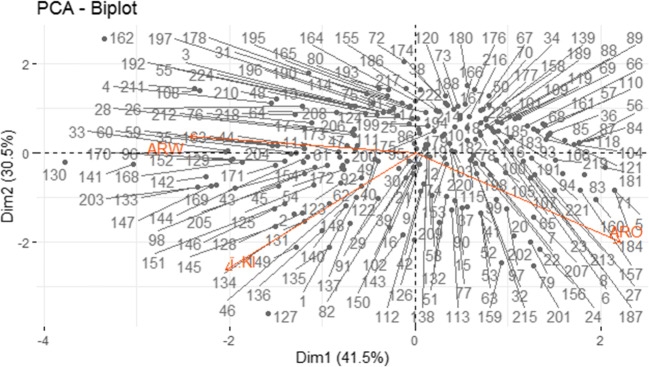
Goat flocks (dots) and reproductive performance measures (light blue) along the first two PCs

In the next step, a PCA was performed combining ARO and ARW together with the remaining annual reproductive performance measures. Significant loadings were identified for kidding interval, PC_ARO_1 and PC_ARW_1 (Table 4). Final PCA was conducted on components that had significant loading in 2nd step and one component with an eigenvalue of 1.24 accounted for 41.5% of the total variance which was used to define the G-ARPI. G-ARPI had positive loadings from ARO and negative loadings for the kidding interval (KI) and ARW. Thus, it reflected the negative relationship between reproductive wastage and annual reproductive output. Figure 2 shows 95% concentration ellipses for each production system and shows that variability among pastoralist herds is smaller than among mixed systems. Figure 3 shows the distribution of the reproductive performance measures and observed flocks along PC1 and PC2.

**Table 4:** Loadings of components from the principal component analysis based on data of 224 dryland flock. G-ARPI accounts for 41.5% of the total variance. Marked in italics are the relevant component loadings with values higher than 0.4

RP measures components	PCA all variables	PCA with only relevant variables
	Component score	G-ARPI
Kidding interval	*− 0.53*	*− 0.53*
Age at first kidding	*−* 0.30	
Reproductive wastage (PC_ARW_1)	*− 0.58*	*− 0.63*
Reproductive output (PC_ARO_1)	*0.54*	*0.57*
Eigenvalue	1.27	1.24
% of explained variability	31.7%	41.5%

*RP* reproductive performance

The calculated G-ARPI values were then transformed to a 10 scale initially by setting the minimum component score value as 1 by adding 4.78 and multiply by 1.4 to expand the distribution up to 10. Details of the final algorithm are presented in the additional file 1.

**Fig. 4:**
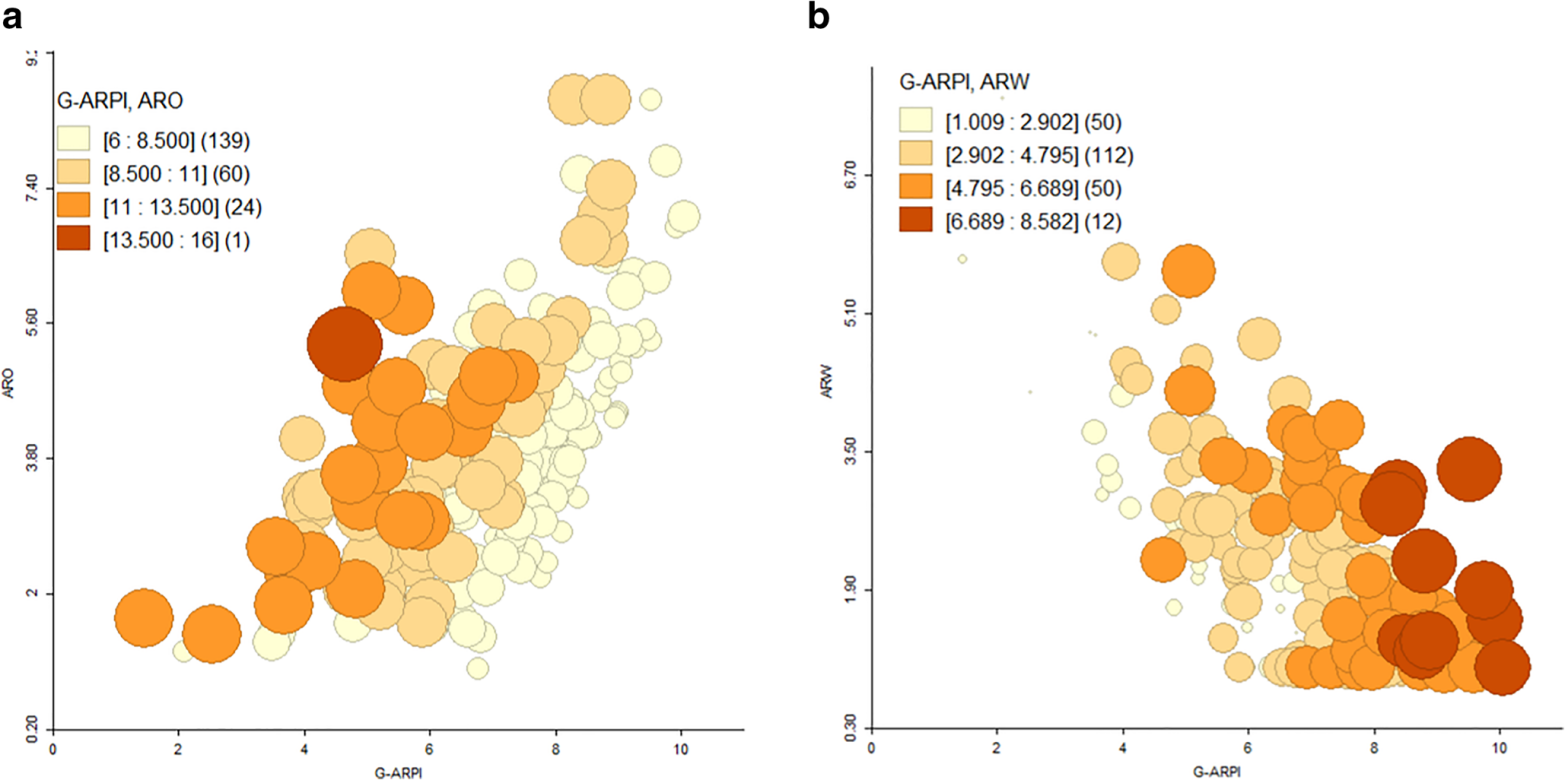
Bubble chart representing the relationship between G-ARPI, ARW and ARO (**a**) and G-ARPI, ARO and KI (**b**) for 224 flocks in dryland of Ethiopia

**Fig. 5:**
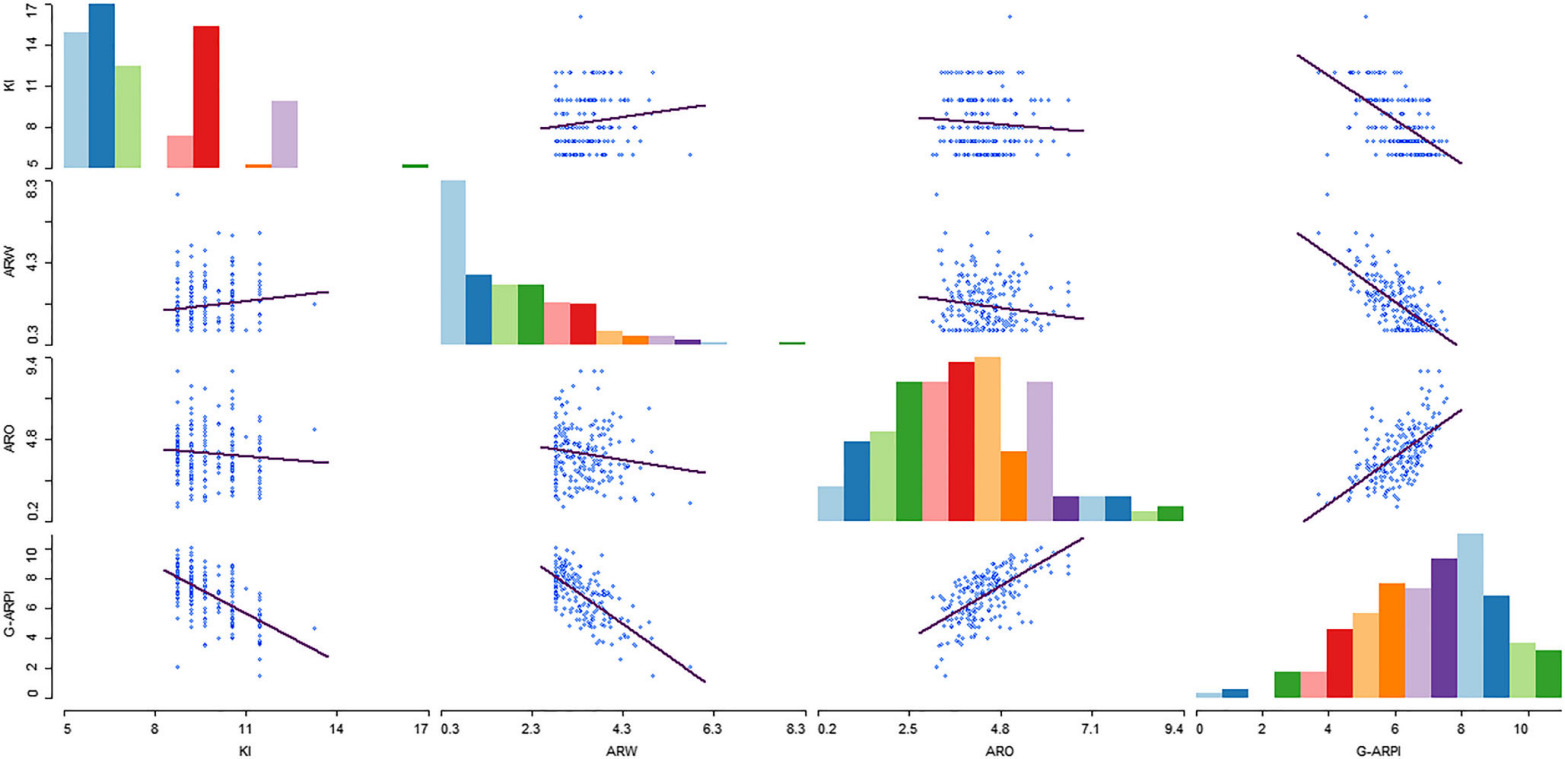
Scatter plot matrix showing the relationship between underlying reproductive performance measures and annual reproductive performance index for 224 flocks in dryland of Ethiopia

**Fig. 6:**
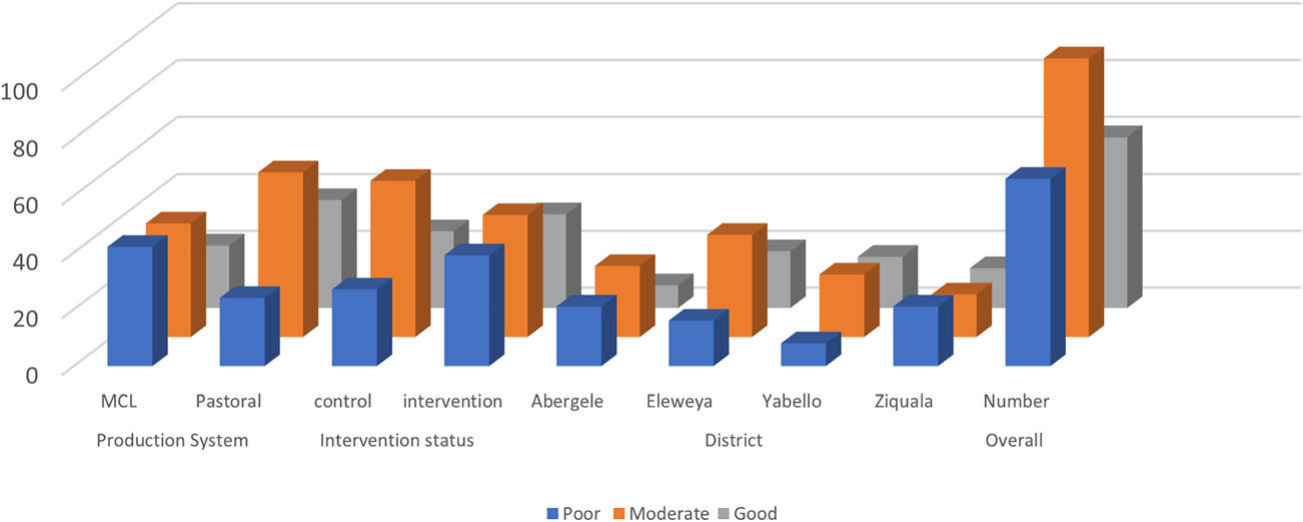
Distribution of reproductive performance categories of goat flocks in drylands of Ethiopia by the production system, intervention status and district

Eighteen observations had to be excluded from analysis due to one or more missing measures. Hence, G-ARPI was estimated for 224 flocks, resulting in a mean G-ARPI score of 6.93. To define performance categories, cut-offs were identified upon visual examination of the bubble charts (Fig. 4). Mean value for underlying reproductive performance measures is shown in Table 5. The scatter plot matrix was used to visualize the relationship between underlying standardized reproductive performance measures and G-ARPI (Fig. 5). Flocks with a G-ARPI above 8.5 (15.63% of the flocks) were considered as good performing flocks, with higher scores for reproductive output measures and lower scores for reproductive wastage, while flocks with an index lower than 6.5 (36.16% of the flocks) were considered as poorly performing flocks. Many of the flocks (48.21%) had a score between 6.5 and 8.5, categorized as moderately performing flocks (Fig. 6). From the 104 flocks studied in mixed crop-livestock lowland production systems, only 13 (12.50%) were classified as well-performing flocks, whereas 44 (42.31%) and 47 (45.19%) flocks were categorized moderate and poor performers, respectively. In the pastoral production system, from a total of 115 flocks, 22 (18.33%) of flocks were categorized as well-performing flocks, whereas 64 (53.33%) and 34 (28.33%) flocks were categorized as moderate and poor performers, respectively (Fig. 6). The result of this study showed that a relatively higher number of flocks (18.26%) from intervention sites categorized as good performed compared with control site flock (12.84%). But the difference is not statistically significant (*p* > 0.05).

**Table 5:** Mean value of underlying reproductive performance measures in dryland goat flocks of Ethiopia

RP categories	ARP index	N	PR	KR	WR	KI	AR	KMR	BDR
Poor	< 6.5	81	71.38	49.04	36.93	9.72	39.33	29.561	18.8
Moderate	6.5–8.5	108	84.62	74.72	63.04	7.62	12.90	13.12	9.11
Good	> 8.5	35	108.20	114.47	107.56	6.88	6.17	7.32	1.58

*RP* reproductive performance, *N* number of flocks, *PR* pregnancy rate, *KR* kidding rate, *RW* weaning rate, *KI* kidding interval, *AR* abortion rate, *KMR* kid mortality rate, *BDR* birth defect rate

## Discussion

Measures of reproduction in goats commonly used include fertility, age at first kidding, kidding interval, kidding rate, weaning rate and kid mortality rate. However, none of these measurements on their own provide conclusive information on overall flock reproductive performance or manages to capture in an objective way levels of performance. For livestock producers, development partners and government initiatives that want to benchmark and compare the performance of flocks, approaches that quantify reproductive performance by combining various aspects of the reproduction process is useful as it allows better monitoring over time. In this study, we present a novel quantitative tool that allows farmers/pastoralists, veterinarians and researchers to determine reproductive performance at flock level. The reproductive performance of the flock was estimated based on the annual reproductive output, kidding interval and annual reproductive wastage. Information used to derive the index was obtained from smallholders’ farmers/pastoralists, and hence, the resulting index can only be valid for the systems included. The difficulty for researchers to obtain a complete understanding of the flocks’ reproductive performance during a single farm visit made the use of interview-based progeny history approaches appropriate. The outcomes of progeny history depend on the memory, perceptivity and reliability of the interviewees. To improve the reliability of data and to reduce recall bias, we used the 1-year memory of famers/pastoralists and enquired confidence of answers. The collected information yielded reliable estimates of reproduction data among the studied flocks and are comparable with reproductive measures reported by various researches conducted elsewhere in Africa (Wilson 1989; Otte and Chilonda 2002; Hary et al. 2003; Dereje et al. 2015). More reliable and precise data could be collected in a longitudinal approach that has shorter in-between visit intervals or approaches that allow ongoing data collection over time, such as for example information communication technology (ICT)-assisted approaches.

Principal component analysis (PCA) is a powerful multivariable technique to identify latent components that are present in a biological process (Wang and Du 2000). It has been used in animal breeding studies to condense the information contained in breeding values and reproductive traits predicted for all available traits into fewer uncorrelated (i.e., orthogonal) latent variables (Boligon et al. 2016). It has been also used to generate variables representing biologically meaningful aspects of variation among qualitative and quantitative morbidity variables related to animal diseases, such as for example to quantify post-weaning multi-systemic wasting syndrome severity at farm level in England (Alarcon et al. 2011). In this study, PCA was used both as reproductive measures dimensional reduction technique and to develop the final model to predict reproductive performance scale.

The presence and high negative loading of reproductive wastage on G-ARPI indicate its important contribution to poor reproductive performance which needs to be addressed appropriately in any intervention plan to increase reproductive performance. This study indicated that the kidding interval appears to be an important descriptor of reproductive performance. The number of young produced per breeding female is of major economic importance (Wilson 1989). One of the most important ways of increasing young produced per breeding female is through reduction of the kidding interval and, if done with optimal input, this may help in meeting the growing demand of the export trade (Abebe 2008).

We normalized G-ARPI to a scale of 10 to facilitate communication and for its use in future projects and to allow comparison over time. Although the establishment of cut-off values in this scale is subjective, clear cut-offs could easily be determined and seemed reasonable. While benchmark figures for reproductive performance in goat flocks in Ethiopia are difficult to find, the values of the underlining measures feeding into the index cut-offs are well aligned with optimum values reported for arid and semi-arid areas of Africa (Wilson 1989; Otte and Chilonda 2002; Hary et al. 2003; Solomon et al. 2014; Dereje et al. 2015).

Flocks presenting a score lower than 6.5 G-ARP index were considered as a poor performer while flocks with a score higher than 8.5 were considered as good performing flocks. Many of the flocks were moderately affected by reproductive failures, consequently categorized as moderately performing flocks. The categorization allowed the identification of extreme flocks, good performer, or poor performer flocks. The presence of a larger number of moderately performed flocks gives opportunities for development partners and smallholders to upgrade those flocks with moderate improvement of managemental practices, feed supplementation and health care. Household modelling in Ethiopia by Mayberry et al. (2018) showed that reproduction, growth and survival rates can be increased through better nutrition and healthcare. Providing improved nutrition by supplementation with low-cost, farm-generated feed resources potentially increase reproductive performance of West African Dwarf goats (Amole et al. 2017). The distribution reflects the severity of reproductive wastage in the Ethiopian dryland goat population. To overcome these challenges, it is important to implement integrated intervention packages which improve feed efficiency, reproduction, parasites and disease control. The presented algorithm can be a useful and objective tool to compare reproductive performance between breeds, management systems, agro-ecological zones and interventions, especially in projects targeting dryland production systems. A future activity should focus to validate and possibly to re-calibrate the index and its cut-offs and to adapt the approach for other production systems in different agro-ecologies.

## Conclusions

The goat reproductive performance index presented here can serve as a basis for analysing reproductive performance and to identify husbandry and environmental factors affecting reproductive performance, study risk factors for reproductive wastage and it will also be used in measuring and monitoring reproductive performance to make flock management adjustments. Ultimately, the index is an ideal management tool to help to evaluate the costs of poor reproductive management which are often hidden and help to the development of economic models that aim to identify the most cost-efficient intervention option to increase reproductive performance. Besides, the G-ARPI can then also be used to monitor the impact of interventions implemented in these goat production systems.

## Supplementary Information

ESM 1 (XLSX 58 kb)
